# Enhanced image‐splicing classification: A resilient and scale‐invariant approach utilizing edge‐weighted local texture features

**DOI:** 10.1111/1556-4029.70143

**Published:** 2025-07-30

**Authors:** Arslan Akram, Muhammad Arfan Jaffar, Javed Rashid, Salah Mahmoud Boulaaras, Muhammad Faheem

**Affiliations:** ^1^ Faculty of Computer Science and Information Technology The Superior University Lahore Pakistan; ^2^ Information Technology Services University of Okara Okara Pakistan; ^3^ Department of Mathematics College of Science, Qassim University Buraydah Saudi Arabia; ^4^ VTT Technical Research Centre of Finland Espoo Finland

**Keywords:** discrete wavelet transform, forgery detection, local binary pattern, multiscale analysis, noise inconsistency detection, pattern recognition, robust feature representation, support vector machine classifier, texture analysis

## Abstract

The spread of image editing tools demonstrates how modern mixed‐media technology enables changes in digital images. Such easy access raises severe moral and legal concerns around the potential for malicious image editing. Overcoming this difficulty will need the development of innovative approaches for the quick detection of changes in high‐quality photographs. This paper proposes a new way to solve this problem by analyzing chrominance discontinuities in spliced regions, DWT, and unique histograms based on local binary patterns. To start extracting the luminance and chrominance components, we change the input image's color space from RGB to YCBCR. Then, using discrete wavelet transformation, the blue and red chromaticity levels were converted into wavelet bands. We compute histograms using the CB and CR DWT high‐frequency bands. The next step is to use feature fusion methods to merge the CB and CR feature vectors from each high‐frequency band after we change the histograms into vectors. Finally, we train a Support Vector Machine (SVM) using the combined color characteristics. A binary SVM trained to identify spliced images between original and spliced images has been produced. Improving upon existing methods, the proposed method achieved up to 98.49% accuracy on CASIA v1.0, outperforming existing benchmarks, that is, 97.33% on DVMM and 98.25% on Casiav2.0, thereby enhancing splicing forgery detection. This method contributes to media forensics by providing a reliable tool for detecting tampered images, which holds significant relevance in legal investigations and digital content authentication.

**Highlights**

- DWT and LBP histograms enhance detection of splicing in high‐quality forged images.
- Novel feature fusion of chrominance components boosts forgery classification accuracy.
- RGB‐to‐YCBCR conversion improves extraction of tampering‐sensitive image features.
- Achieved over 98% accuracy on benchmark datasets for splicing forgery detection.
- Proposed SVM‐based model offers a scalable, efficient solution for forensic image analysis.

## 1 INTRODUCTION

The internet, newspapers, the armed forces, and security cameras are just a few examples of the various modern‐day applications of photography. Because contemporary image alteration programs can change an image's content, photo confirmation [1] could become a big problem [2]. Problems, including misleading advertising, falsification, dark mailing, and extortion, have emerged due to photo editing. Images must undergo independent verification before being utilized as evidence in a court of law or as a source of information. Picture imitation, especially when done intentionally, sometimes involves copy–move and splicing techniques. Photo editing tools like fraud preparation make merging or using similar images to create new ones easy. Copy–move and splicing involve slicing and pasting images into the original, two picture manipulation methods [3]. The synthetic locations are subjected to several post‐processing (PP) procedures to conceal the imitation cues, including scaling, obfuscation, noise, compression, and turning. Picture sensors and mobile phones are essential in mass‐producing digital photos, but they have also led to the emergence of image forgeries, particularly in the splicing technique [4].

Several algorithms [5, 6], which may be dynamic and detached, were developed to detect applying forgery based on the wavelet components [7, 8]. To verify its authenticity, dynamic strategies eliminate this watermark or signature. The premise of dynamic techniques is that the authenticity of a given image is guaranteed by including data, such as an imprint or signature [9], at the time of purchase [7]. These strategies have very limited applicability because, in most cases, data is outside the watermark or signature. Because of this limitation, passive methods that do not rely on data for grafting fraud detection have been developed [8]. Changing the content of one image by duplicating and superimposing its content onto another is what picture joining is all about. Joining is an important and popular method of photo fraud. The public's trust in automated photo verification relies on accurate picture joining, making this an increasingly important field of investigation for computerized image fraud. The image grafting process diminishes the material's consistency, smoothness, and uniformity. These details are crucial when spotting the deceitful areas in the picture. Modern picture grafting systems consider the numerous worldwide factual factors shown by unanticipated irregularities in grafted images.

Partitioning the input image into sub‐bands using DWT is the initial step in identifying the joining‐induced image discontinuities. All four wavelet sub‐band coefficients are fairly communicated with by the DWT's robust de‐correlation capabilities. This study suggests a new way to tackle the problem by using image changes, DWT, and special histograms that highlight important patterns in images. We start by converting the YCbCr input picture to RGB before we can extract the chrominance and brightness components. Then, using discrete wavelet transformation, the blue and red hues were converted into wavelet bands. We compute histograms using the CB and CR DWT high‐frequency bands. The next step is to use feature fusion methods to combine the CB and CR feature vectors from each high‐frequency band after changing the histograms into vectors. The final step is to train the model to tell apart real images from altered ones. This is done by merging the color features and using a support vector machine (SVM) for the training. The bold goals and potential to substantially improve image alteration detection made possible by this study constitute a paradigm shift.

- Develop a method for accurately classifying splicing forgery using discrete wavelet transformation and DRLBP histograms. The work utilizes support vector machines (SVMs) for picture classification based on extracted features and DWTs and DRLBPs for optimal feature extraction from benchmarked splicing datasets.
- The suggested method should be tested on various benchmark datasets, such as CASIA (1.0), CASIA (2.0), DVMM, and the combined dataset, in order to assess its effectiveness and robustness.
- Test how effective the proposed method is with different settings, like changing picture sizes, compression rates, and noise levels, especially for images that have many fake parts.
- Using metrics like true positive rate and false positive rate, compare the suggested method to current state‐of‐the‐art procedures to show that it is better.

Section 2 provides a concise overview of relevant publications, with an emphasis on the examination of patterns in scientific literature. The methods of data collection are detailed in Section 3. Section 4 presents a detailed analysis and the study's findings. Section 5 compiles the research, highlights key findings, and provides suggestions for future studies.

## 2 RELATED WORK

The vast majority of methods exist for passively or unthinkingly joining fake sites [10]. Alahmadi et al. [11] and Begum and Uddin [12] utilized DCT coefficients, least and most extreme channels, to identify grafting fraud and extract highlights from picture squares. Many calculations employ multi‐resolution methods such as DWT [13, 14]. To detect grafting fraud, filter highlights are utilized instead of piece coordination. Many methods for discovering joining imitations are evaluated using datasets from CASIA (v2.0), Columbia Color (CC) DVMM [15], and CASIA (v1.0) [16]. Recent studies have developed classic fake picture detection algorithms based on machine learning to classify splicing forgeries in photos. Jalab et al. [17] have introduced a straightforward machine learning system that utilizes a hybrid feature extraction method, HoG and GLCM. The HoG algorithm enables the extraction of texture patterns from photographs. On the other hand, the GLCM algorithm relies entirely on statistical patterns seen in photos. The GLCM technique is employed to enhance the performance of the HoG method. The method is inferior compared to outdated methods but outperforms them using CASIAv2 splicing image database. Accuracy, True Positive Rate, and False Positive Rate are the metrics employed for evaluating performance. The approach has a high accuracy rate of 97%, making it suitable for detecting spliced images. The primary constraint is using a single dataset for experimentation, resulting in concerns over generalizability and reliability. Furthermore, feature reduction is detrimental as it may result in the loss of valuable characteristics, ultimately leading to a drop in performance.

On the other hand, [18] demonstrated the use of 3D image moments to detect image grafting, while [19] introduced support vector machines utilizing on‐camera response functions. We used 2D and 1D moments, Markov chain probabilities (MCP), and DCT coefficients to look into how to find image grafting. Experiments conducted on the CASIA (v2.0) dataset achieved an accuracy of 84.86%. He et al. [8] got even better results, up to 89.76%, by combining SVM with MCP, DCT, and DWT coefficients. Their results were better than Shi et al.'s. In another study, MCP was taken from the Cb channel to find possible spliced images. It worked 95.49% of the time on the Columbia Color (CC) dataset and 89.24% of the time on a subset of CASIA (v2.0). Also, Zaho and Tian [20] improved the method Wang et al. [18] came up with by using a chrominance channel to tell the difference between grafted images and the first method. Zhang et al. [21] introduced a conventional machine learning methodology that utilizes LBP and Error Level Analysis (ELA) to classify splicing forgeries. LBP features were used for classification using SVM. The experimental procedure utilized the Columbia dataset, demonstrating an accuracy rate of 91.46%. The method is effective at classifying splicing images; however, it is occasionally limited by poor performance and disregard for generalizability due to using a single dataset in the experiments and the absence of testing on unseen samples.

Examining the growing issue of online image counterfeiting, Barin et al. propose a feasible countermeasure. A Standard Deviation‐Local Binary Pattern (SD‐LBP) splicing detection system and an ANN classifier were used in their method. The SD‐LBP makes things more reliable by using SD value‐based thresholding. The ANN classifier facilitates the obtaining of the optimal accuracy from features. With a 97.8% accuracy rate on the CASIA V2.0 data, our approach beats existing ones. K. Sudhakar et al. stress even more the increasing concern about picture counterfeiting and the need for advanced detection methods. Despite the exploration of CNN models, many CNN‐based methods only detect specific types of counterfeiting. Their work presents a lightweight deep‐learning method with 92.23% validation accuracy to detect double image compression frauds. The approach offered a beneficial way to handle misleading visual content and beats present standards. Using fundamental forging techniques on digital photos taken by Internet of Things (IoT) devices, Islam et al. tackle cybercrime. We need to evaluate how well current models handle minimal editing tools and distorted photos from IoT sensors. DCT and LBP worked better together for feature extraction and detection. With a peak accuracy of 96.14% [22], this method outperformed top algorithms on numerous datasets, including an IoT‐based forgery dataset.

Image forgery detection literature abounds with approaches and datasets that promise to make the process more efficient and accurate. By integrating textural information with support vector machines (SVMs), researchers could attain a remarkable 98.40% accuracy for authentic and spliced photo classification on the CASIA v2.0 dataset. Additionally, they reached a commendable TPR of 98.80% and a TNR of 98.00%. Using DRLBP characteristics along with SVM classification has made a big difference in the field across a number of datasets, such as DVMM, CASIA v1.0, and CASIA v2.0 [17]. The approach proved strong with an incredible 98.95% detection accuracy for both real‐world and tampered photos. A new study [23] looked at deep‐learning techniques using a CNN architecture with four convolutional layers and max‐pooling. On the CASIA v1.0, CASIA v2.0, and CUISDE datasets, this model did pretty well with a 99.3% difference between real and spliced images. For digital image‐splicing and copy‐paste forgery detection, a coherent method was devised by Agarwal et al. [24]. Extracted utilizing the DCT for splicing detection and Accelerated‐KAZE (AKAZE) features for copy‐paste forgery detection, the method combines texture‐based Orientation Invariant Local Binary Pattern (OILBP) features. Standard datasets, including Extended IMD2020, CASIA v1.0, and CASIA v2.0, were used to evaluate the suggested technique, obtaining an overall accuracy of 98.87% for splicing forgery detection and 96.50% for copy‐paste forgery detection. This method is more complete than others and is limited to a single form of fraud since it can concurrently identify two kinds of forgeries, therefore, addressing two aspects of them. Nevertheless, the method's dependency on high computer resources resulting from the intricacy of feature extraction and classification procedures could restrict its real‐time use.

Alternatively, [25] achieved a remarkable 98% accuracy rate for recognizing spliced and real images using SVM classification with factors like entropy, LBP, skewness, and kurtosis on the CASIA v1.0 and CASIA v2.0 datasets. With the CASIA and COLUMBIA datasets, the authors of [26] aimed to improve mobile‐based identification and achieved an accuracy rate of 96.4% when separating real images from spliced ones. A study on region‐based localization algorithms discovered that CRPN and MFPN performed well on synthetic datasets such as NIST16, CASIA 1.0, and CASIA 2.0 [20]. With an average precision (mAP) of 93.4%, their single‐spliced area localization achieved significant strides in targeted region recognition. Despite the lack of measurable results, [27] presented DDRU‐NET for localizing single‐spliced regions on the CASIA V2.0 and DEFACTO datasets. The MISD, CASIA v1.0, Wild Web, and Columbia datasets were used by [28] to localize single‐spliced areas using MobileNet V2 combined with MRCNN. With an accuracy of 82%, they were useful in detecting altered regions in images. Various methodologies and datasets are covered in this research, contributing to the ongoing improvement in photo forgery detection. The accuracies vary, and the detection process is focused on different aspects. A new image‐splicing detection method using Otsu‐based enhanced local ternary pattern (OELTP) and frequency‐based edge‐texture characteristics was presented by Srivastava and Yadav [29]. The approach detects essential edges in the image using Canny edge detection and then applies multi‐level discrete wavelet transforms (DWT) for digital image filtering. These margins then maximize the Otsu global threshold, therefore, optimizing the OELTP feature‐extracting technique. The method was tested using three publicly available datasets: Columbia, CASIA v1.0, and CASIA v2.0. With accuracy rates of 99.98% on CASIA v1.0, 99.03% on CASIA v2.0, and 98.56% on the Columbia dataset, the proposed approach yielded shockingly good results. The method performs well in forgery detection but suffers when background lighting is quite non‐uniform, implying that further treatment of such complicated issues needs to be improved. Emphasizing the use of enhanced local feature extractors paired with ideal classifiers like Support Vector Machine (SVM) and Naive Bayes Classifier (NBC), a novel approach for copy–move forgery verification in images was developed by Babu and Rao [30]. The method employs feature extractors like Local Ternary Pattern (LTeP), Local Phase Quantization (LPQ), and Local Gabor Binary Pattern Histogram Sequence (LGBPHS) to capture minor changes in texture even under demanding conditions such as JPEG compression, scaling, and rotation. The proposed algorithms were assessed on many datasets, including CoMoFoD, CASIA, and MICC, achieving high accurate positive rates (TPR) and low false favorable rates (FPR), with the best performance metrics found when LGBPHS was paired with an optimal SVM classifier. Still, the complexity of the approach and computing needs could make real‐time applications challenging.

In today's world of advanced computing and computerized images, the most crucial duty for the secure and authentic transfer of mixed‐media content is the identification of photo impersonation. Photo graft placement was determined by Alahmadi et al. [31] using DCT and LBP characteristics. To differentiate grafting‐induced picture abnormalities, Pham et al. [32] employed Markov highlights extraction. For data classification, SVM was employed. Jalab et al. [17] employed SVMs for classification after extracting fragmentary entropy from DWT coefficients. Skillet developed a practical method for identifying copied areas within a picture frame. The proposed method locates the identically changed spots by locating the critical points utilizing geometric changes. To find the best spots to insert image grafts, Wang et al. [18] came up with a novel deep‐learning approach. The suggested CNN learns a weighted combination of three distinct kinds of highlighted extraction algorithms. Convolutional neural networks (CNNs) discover the optimal parameter sets for feature extraction methods. Alsandi [33] presented a passive splicing detection method based on the Mean‐LBP for texture analysis and the Speeded Up Robust Feature (SURF) descriptor. The method consists of three stages: SVM classification, mean‐LBP feature extraction, and SURF's ROI extraction. Analyzed on datasets comprising CC, CASIA TIDE V1.0, and CASIA TIDE V2.0, the proposed approach obtained corresponding high accuracy rates of 97.9%, 98.2%, and 98.9%. The method excels in identifying spliced sections; handling highly compressed images and other post‐processing methods still challenges us.

## 3 MATERIALS AND METHODS

**FIGURE 1:**
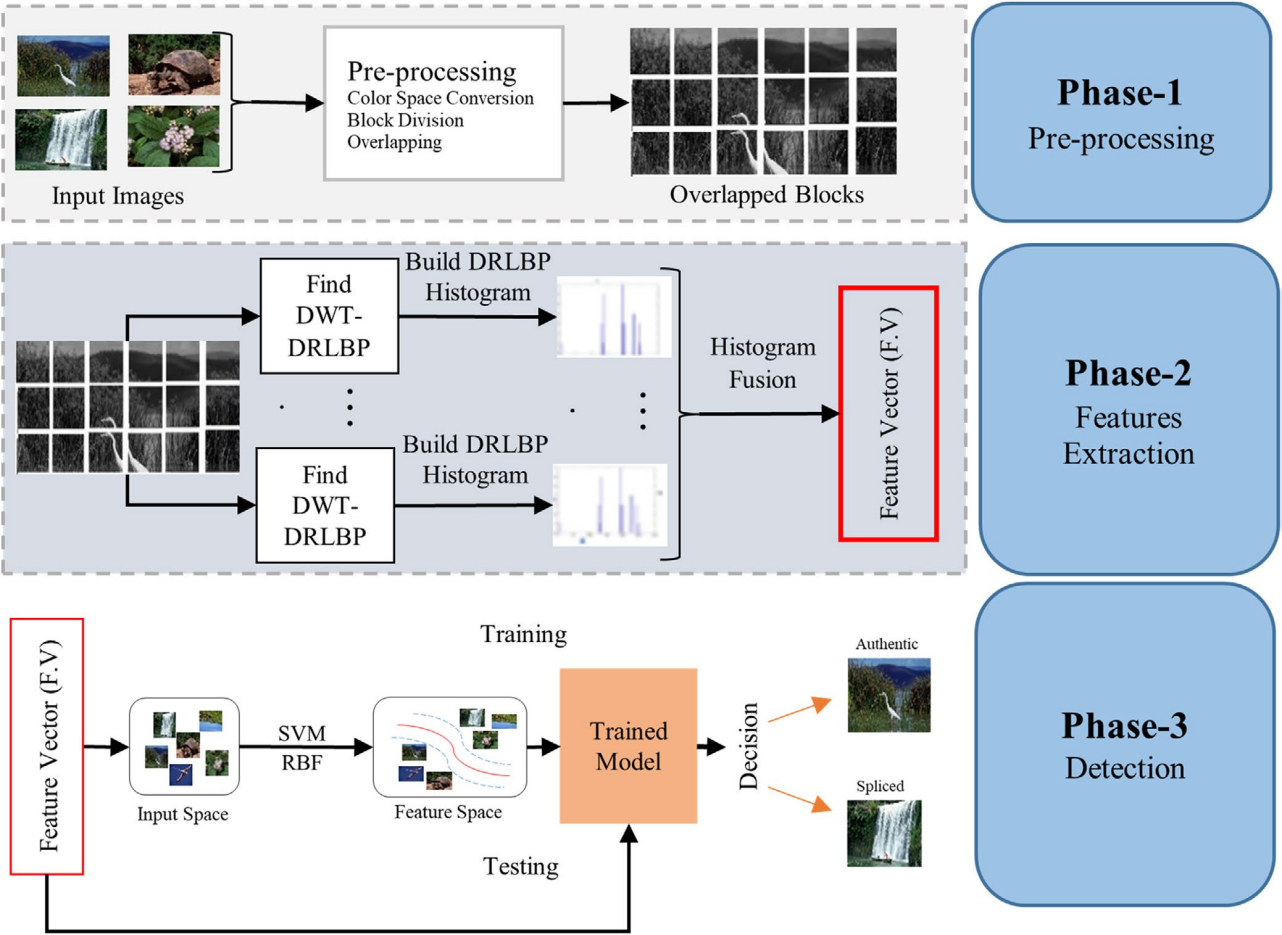
Architecture of proposed splicing forgery detection approach.

In the section, the proposed method has been described in detail. Proposed methodology can be categorized into different steps. Firstly, the input image has been converted from RGB color space to YCBCR to get the luminance and chrominance components of the input image. Next, the Chrominance of blue and the Chrominance of red have been transformed into wavelet bands using discrete wavelet transformation. Histograms are calculated from DWT high‐frequency bands of CB and CR. Then, histograms are converted to feature vectors, and feature vectors CB and CR from each high‐frequency band are fused using feature fusion methods. Finally, fused features of each Chrominance have been concatenated and fed to a Support Vector Machine (SVM), which trains the classification model to classify authentic and spliced images. We evaluate our suggested approach using open‐source benchmark datasets to show its scalability and applicability. Multiple splicing datasets have been used to evaluate the proposed method. Following a one‐level DWT connection inside the picture, the DRLBP [3] description is appended. Before classification, the DRLBP feature vectors were normalized using min–max normalization to scale the values to the range [0, 1], ensuring uniform feature contribution to the SVM model. Figure 1 shows the recommended splicing image classification architecture.

### 3.1 Datasets

To evaluate the image detection method, we used three freely available benchmark datasets: CASIA (1.0) [16], CASIA (2.0)—to assess the image detection technique, and Columbia Color DVMM [3]. We first assessed the suggested approach using the DVMM dataset. For further testing and assessment, we advise researchers to take advantage of the CASIA (1.0) and CASIA (2.0) datasets. The DVMM collection consists of 183 real TIFF images and 180 modified ones. The 1.0 CASIA dataset comprises 801 authentic and 921 manipulated photos. Every handled image receives particular geometric modifications. Table 1 shows that the 7491 photos in the CASIA (2.0) dataset consist of 5123 legitimate ones. We also evaluated the image forgery localization approach on a combined dataset comprising the three datasets we just mentioned in order to check whether it might be applied in other circumstances.

**TABLE 1:** Benchmarked datasets used for experimentation.

Sr.	Dataset	Authentic	Spliced	Total
1	CASIA (1.0)	800	921	1721
2	CASIA (2.0)	5123	7491	12,614
3	DVMM	183	180	363
Total		6106	8592	14,698

### 3.2 Preprocessing

**FIGURE 2:**
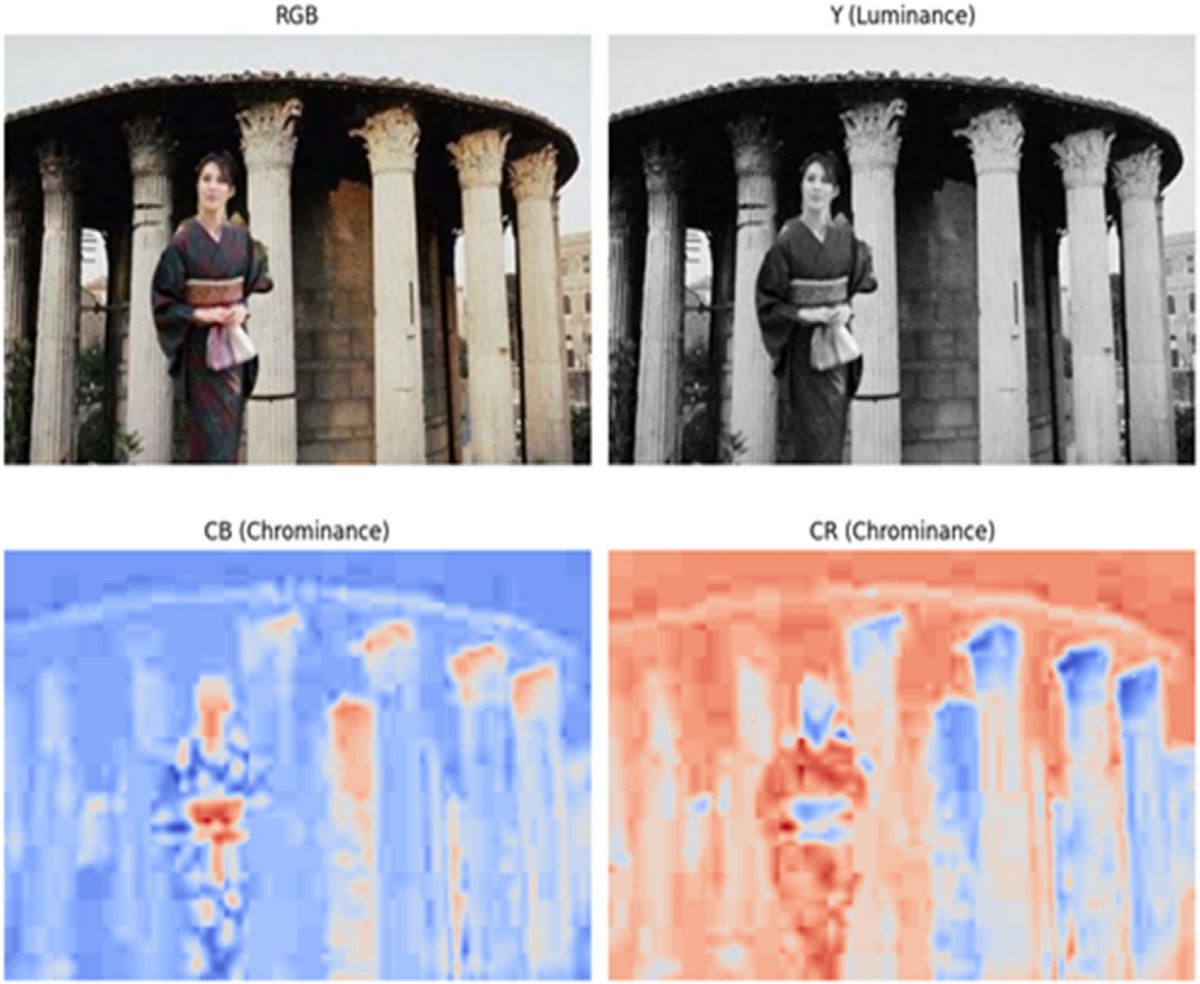
Y, Cb, and Cr channels of an RGB image after conversion to the YCbCr color space.

The adjusted image is displayed in Figure 2 using the YCbCr color scheme, where Y denotes the brightness component and Cb is the chroma component. The brightness channel can conceal material changes by recording the image's content. Forgery alters surface patterns, resulting in abrupt corners, lines, and edge transformations. The CB and CR chrominance channels store the red‐difference chrominance and blue‐difference chrominance data, respectively. These channels are particularly useful at detecting small color issues that can occur when images are stitched together, such as strange color variations and artificially sharp edges, which would otherwise be invisible in the (Y) channel. The two chrominance channels will be examined independently of each other, and this will allow the technique to have its more detailed and distinct characteristics, thus contributing to the agent's ability to find more accurate forgeries. An image's chroma components aid in identifying surface imperfections such as corners, lines, and edges because they convey subtle signals. Forgery uses DWT components to effectively disclose gaps in the edge detection process in images, affecting the local composition of linked images. High‐frequency wavelet groups thereby highlight the modifications introduced by grafting.

### 3.3 Feature extraction

The proposed DWT‐DRLBP descriptor can be used to investigate these modifications; it employs the Discrete Wavelet Transform (DWT) to partition an image's chroma components (CC) into smaller bands and then applies the DRLBP surface signifier, a potentially effective surface descriptor, to these smaller bands.

#### 3.3.1 DWT decomposition of chroma component

**FIGURE 3:**
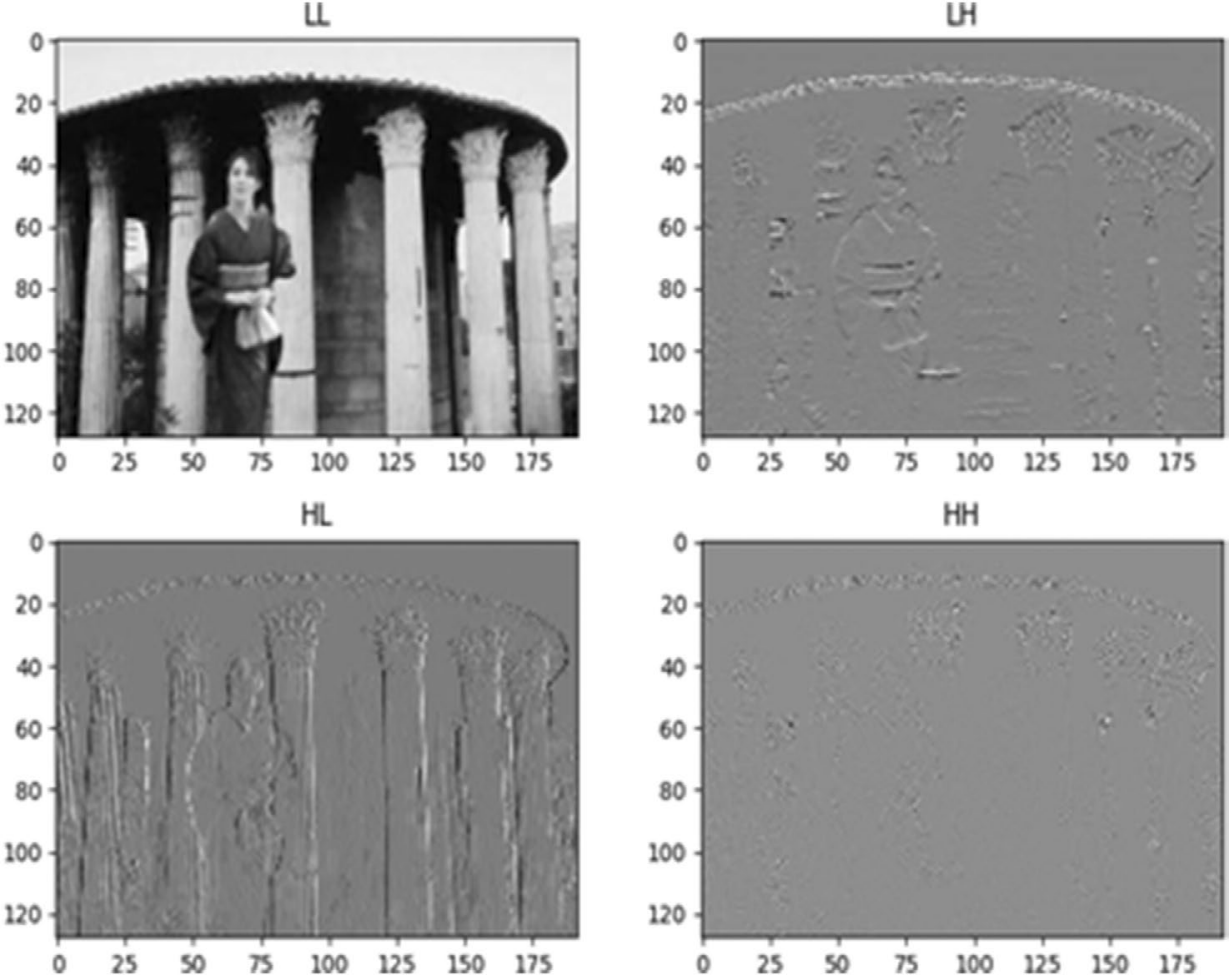
Decomposition of an image into wavelet frequency sub‐bands: LL (Low–Low), LH (Low–High), HL (High–Low), and HH (High–High), capturing different spatial and frequency characteristics for analysis.

Efficiently evaluating the picture shallow with severe motion and most minor wavelet elements is made possible by DWT's exclusive and unfair image. The wavelet factors successfully highlight the underlying differences in picture grafting. In picture joining, the moo recurrence coefficients provide the tall differentiation. The moo recurring features are applied meticulously to the test image. The picture representation approach becomes extremely easy when energy compression is represented using a minimal number of wavelet coefficients. The moo recurrence components draw attention to the picture's imperfections to improve the picture approximations further. Figure 3 shows that when pinpointing changes in image content, low recurrence is the way to go. The encoding of wavelet transformation at the picture grafting point enables using the low‐pass and high‐pass channels to study the image in the frequency domain. Grafting fraud to conceal lines, corners, and edges results in tall repetition instead of tall difference. The wavelet transform defines these shifts using targeted precision and refinement in the recurrence coefficients. Based on these factors, each image's CC is categorized into four recurrent groups: LL, HH, LH, and HL. This classification can better describe grafting‐induced alterations (refer to Figure 3).

#### 3.3.2 DRLBP histograms construction of sub‐bands

**FIGURE 4:**
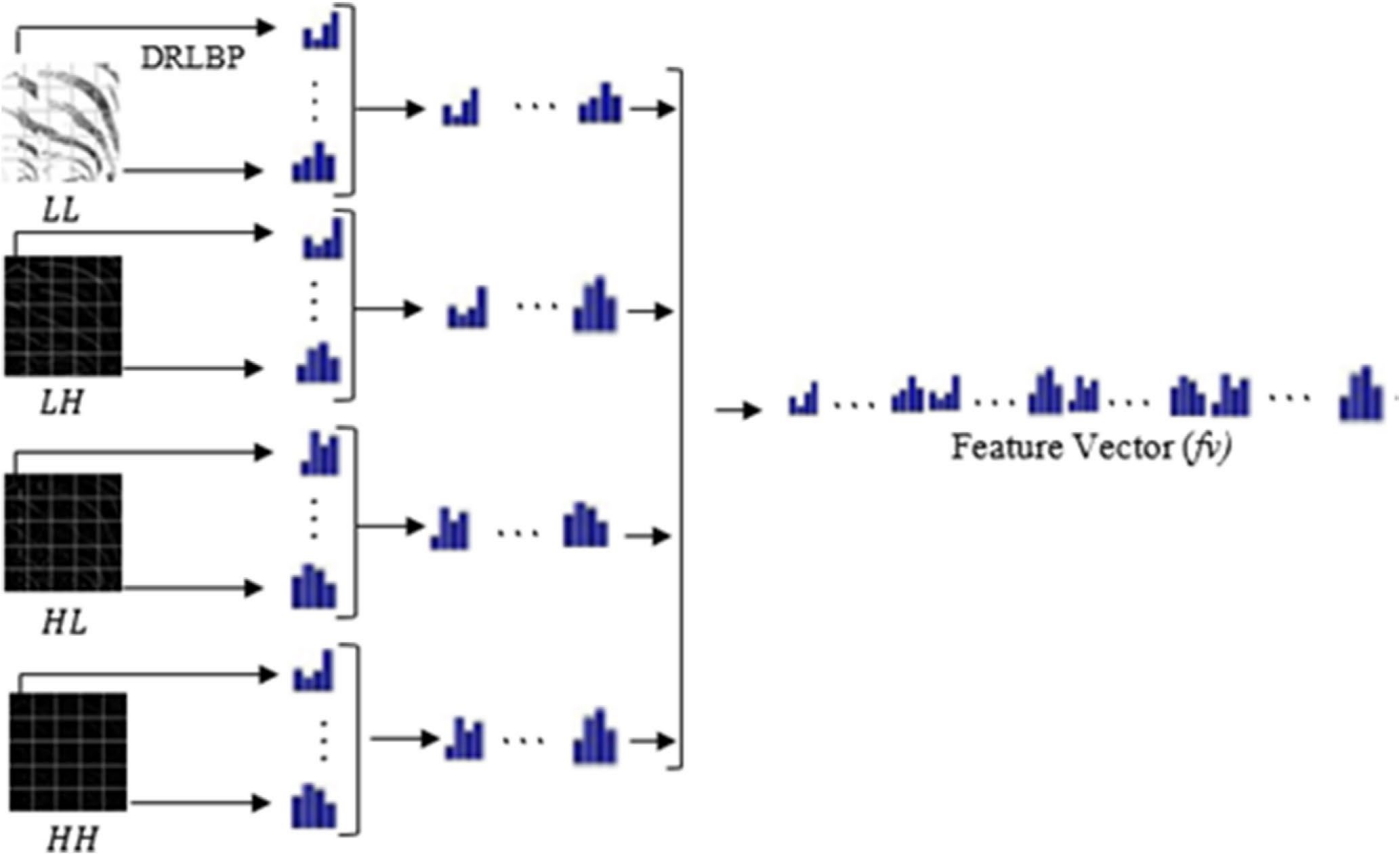
DRLBP histogram visualization across all discrete wavelet transform (DWT) band.

The first step in extracting and assessing the distribution of the distinct patterns from the test image is removing the chrominance components. Finally, we have the DRLBP signifier, which distinguishes the context of LBP codes from the HSG of neighborhood twofold patterns, including edges, dots, lines, and corners [34]. Following that, given the size of the adjacent slope at various sites, we made reasonable estimations on how to implement these ideas. The DRLBP description stresses local changes even while considering an overall change. Although [3] presents DRLBP in all, Equations (1), (2), (3), (4) offer the DRLBP descriptor system. Equation 3 computes the weighted histogram of repeated designs for every pixel in the picture. The two designs are then discovered using a 3 × 3 window with eight neighbors. The 3 × 3 Local Binary Pattern (LBP) neighborhood was applied in this research since it provides a good tradeoff between the speed of computation and local texture sensitivity. This geometry utilizes 8 neighboring pixels to encode texture, effectively capturing any minor variations introduced by splicing. Moreover, by comparing 3 × 3 with larger neighborhoods like 5 × 5 and 7 × 7, we discovered that 3 × 3 had uniformly high classification accuracy and lower computational cost. Related works have also successfully utilized similar configurations, which further supports our decision (Figure 4).

(1) $$W_{\mathrm{LBP}}\left(i\right)=\sum_{m=0}^{S-1}\sum_{n=0}^{T-1}G_{m,n}\delta \left({\mathrm{LBP}}_{m,n},i\right)$$

(2) $$\delta \left(j,i\right)=\left\{\begin{array}{c} 1,\\ 0, \end{array}\right.\begin{array}{c} j=i\\ \;0\;\text{otherwise} \end{array}$$

Equations (1) and (2) define the process of computing a weighted histogram of Local Binary Pattern (LBP) codes across an image or its transformed sub‐band. In Equation (1), $W_{\mathrm{LBP}}\left(i\right)$ represents the weighted histogram value for the LBP code *i*, calculated by summing over all pixel positions (*m*, *n*) within the image dimensions S (rows) and T (columns). Here, $G_{m,n}$ denotes the gradient or importance weight at each pixel location, and ${\mathrm{LBP}}_{m,n}$ is the LBP code at that location. The function $\delta \left({\mathrm{LBP}}_{m,n},i\right)$, defined in Equation (2), serves as an indicator function that returns 1 if the LBP code at (*m*, *n*) matches *i*, and 0 otherwise. This formulation allows the construction of a feature histogram that captures both the frequency and the spatial significance of specific LBP patterns within the image. The image is communicated using *n* = 28 neighbors with 256 individual histograms. The focal point of pixel‐wise neighboring alters is indicated by the angle greatness of the center pixel (*x*, *y*), G (*x*, *y*), which is part of the comparing twofold design. The identification of each wavelet band is indicated by ST. We constructed the weighted histogram W RLBP and eliminated the effect of the turnaround in the frontal and foundational areas by following these methods. Following the computation of the RLBP, the following steps were taken using a computer to enhance the discriminatory impact of the double designs:

(3) $$\text{WDLBP}\left(i\right)=\left|\text{WLBP}\left(i\right)-\text{WLBP}\left(2^{8}-1-i\right)\right|,0\leq i\leq 2^{7}$$

Equation (3) defines the Weighted Difference Local Binary Pattern (WDLBP) histogram value for bin i, represented as $\text{WDLBP}\left(i\right)$. It is computed by taking the absolute difference between the weighted LBP histogram values at index iii and its complementary index $2^{8}-1-i$, that is, $\text{WLBP}\left(i\right)$ and $\text{WLBP}\left(2^{8}-1-i\right)$, assuming an 8‐bit LBP code. The range $0\leq i\leq 2^{7}$ (i.e., 0–127) ensures that only the lower half of the symmetric LBP histogram is considered, reducing redundancy. This operation highlights asymmetry in texture patterns, which can be indicative of tampering in forensic analysis. The DRLBP descriptor is generated by merging each neighborhood location's biased RLBP and biased DLBP histograms as shown in Equation 5. The calculation for this descriptor is as follows:

(4) $$\text{DRLBP}=\left[W_{\text{RLBP}},W_{\text{DLBP}}\right]$$

#### 3.3.3 DRLBP histogram vector fusion

**FIGURE 5:**
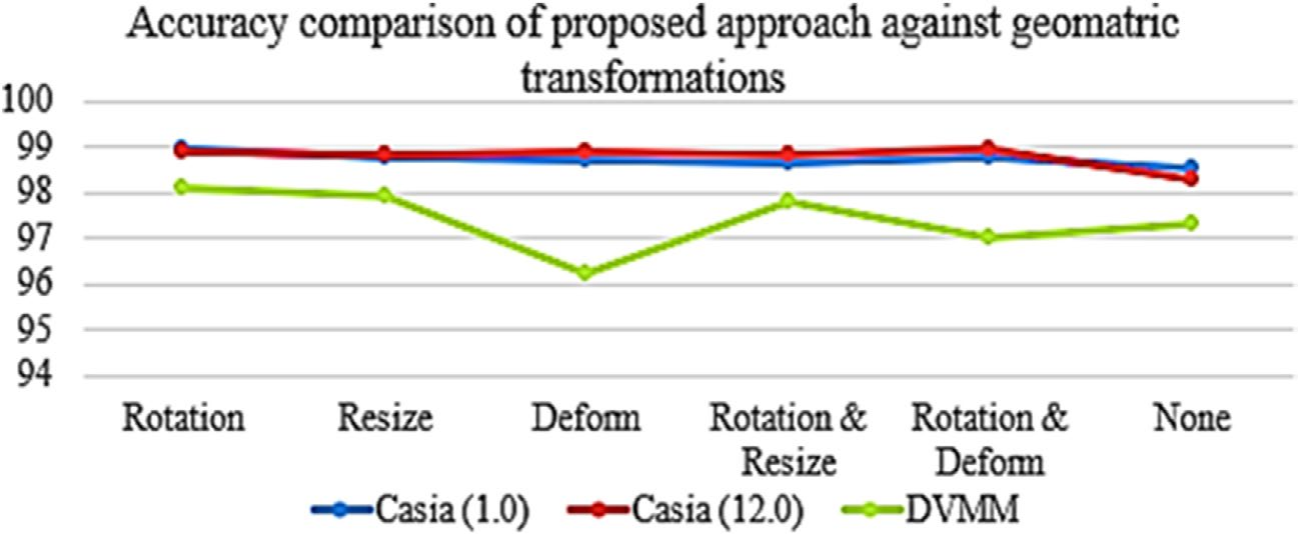
Accuracy comparison of the proposed method in handling post‐processing attacks.

After finding the most similar DRLBP descriptor for each channel (Ch) across all sub‐bands (sb), the HOG features of all sub‐bands are added together to make the DRLBP descriptor. Figure 5 illustrates the complete computation along with its explanation. The descriptor primarily captures auxiliary changes without considering the image's spatial context. Still, adding discriminative information and local spatial changes improves its feature‐distinguishing power. For every channel in this work, we split the test image into S subblocks. With s × t dimensions for every sub‐block, S(s × t) = S × T is the overall dimension. We derive the related descriptor B_i for every sub‐block Bi.

(5) $$\begin{array}{c} {\mathrm{fv}}^{\mathrm{ch}}=\left[{\mathrm{fv}}^{\mathrm{LL}},{\mathrm{fv}}^{\mathrm{LH}},{\mathrm{fv}}^{\mathrm{HL}},{\mathrm{fv}}^{\mathrm{HH}}\right] \end{array}$$

The blue‐red color‐based histograms data of DWT high‐frequency bands combined with the CB histogram information completes the feature extraction phase. These concatenated characteristics enclose credentials of immediate texture and effective data important in the exclusive consequence of genuine areas with disintegrated areas. The second step will be making use of these complete feature vectors to learn an effective classification model, as explained next.

### 3.4 Classification

There are numerous reasons why Support Vector Machines (SVMs) excel at binary classification tasks. The ability of support vector machines (SVMs) to process high‐dimensional data is among their numerous advantages. Before classification, the DRLBP feature vectors were normalized using min–max normalization to scale the values to the range [0, 1], ensuring uniform feature contribution to the SVM model. To mitigate the potential for overfitting, support vector machines (SVMs) optimize the margin between classes to determine a decision boundary that remains effective even after being updated with new data. Support vector machines (SVMs) exhibit consistent performance on test data, rendering them highly suitable for handling sparse or chaotic training datasets. SVMs are frequently utilized in binary classification tasks due to their effectiveness and resilience. SVMs are highly effective instruments for machine learning and data mining by virtue of their robustness and precision in classification. [19, 35].

### 3.5 Parameters tuning

The suggested framework has several parameters. It is an optimization difficulty since thoroughly adjusting the constraints to reach the optimum set is challenging. In practice, parameter setting is essential. In this post, we chose numerous parameter values depending on observations. The suggested method applies the following parameter modification. SVM parameter adjustments are grounded on the produced dataset. We obtained the best bit execution in RBF. The RBF channel requires consistent gamma and coefficient. The ideal mix of these settings defines RBF channel performance. The regularized coefficient alters performance complexity by reaching the maximum imitation location precision. The gamma parameter of the RBF component describes the nonlinear mapping between two focuses, yet the farthest missing focuses are regarded as the nearest when gamma is low. Grid search with the following settings helped to establish the picture grafting position: RBF bit set to 25, regularized constant, and gamma set to 2–5.

### 3.6 Evaluation policy

For 10‐fold cross validation, we used random guesses to divide actual and fake photos into ten equal halves. The framework's performance is shown by the standard deviation of the approximately ten execution degree numbers. Every dataset uses the same procedure. Produced photographs are better for appraisal than real photos. Following execution, we evaluated exactness, affectability, specificity, and false positive rate. Accuracy, which can be computed as follows, is the proportion of tests that are true to all test images or accurately predicted to be produced:

(6) $$\begin{array}{c} \mathrm{ACC}=\frac{\left(\mathrm{TP}+\mathrm{TN}\right)}{\mathrm{TP}+\mathrm{TN}+\mathrm{FN}+\mathrm{FP}}\times 100\%. \end{array}$$

The classifier is anticipated to be constructed based on the total number of tests generated, where “TP” stands for that. If the “TN” value exists, the classifier could correctly recognize authentic images. The classifier's percentage accuracy and authenticity rates for false photographs are displayed in the “FN” and “FP” pictures, respectively.

(7) $$\begin{array}{c} \mathrm{TPR}=\mathrm{SN}=\frac{\mathrm{TP}}{\mathrm{TP}+\mathrm{FN}}\times 100. \end{array}$$

A true negative rate, sometimes called specificity, is the rate at which one assumes that an authentic picture is authentic. This is how it is computed:

(8) $$\begin{array}{c} \mathrm{TNR}=\mathrm{SP}=\frac{\mathrm{TN}}{\mathrm{TN}+\mathrm{FP}}\times 100. \end{array}$$

The erroneous positive rate is the frequency with which test images mistakenly recognized as authentic are anticipated to be generated.

(9) $$\begin{array}{c} \mathrm{FPR}=\left(1-\mathrm{TNR}\right)\times 100\%. \end{array}$$

## 4 RESULTS AND DISCUSSION

MATLAB R2024a and its related tools helped us to build and test the proposed model. The results of an extensive set of tests conducted on the proposed method to evaluate its steganalysis performance are presented in this part. The performance evaluation findings are derived from several evaluation criteria—accuracy, FPR, FNR, etc.—where our approach starts with a non‐overlapping block partition used. Furthermore, feature extraction and classification learning were conducted in MATLAB, which helped with SVM classification. We followed the assessment method suggested in the preceding section for both SVM model training and evaluation. These fields were used for the experiments:

- Assessing the suggested method's efficiency using several benchmarked splicing datasets.
- Comparing the suggested framework's performance with various post‐processing attack models.
- A comparison between the proposed method and the most recent methods in the field.

### 4.1 Performance analysis of the proposed method among benchmarked datasets

Table 2 displays the performance metrics of the proposed approach after training the model on several benchmark datasets. Table 2 displays image‐splicing classification testing results for several datasets using the Support Vector Machine (SVM) model. The table displays the performance metrics for model accuracy, TPR, FPR, and area under the curve (AUC). The findings demonstrate the model's exceptional performance across all datasets. The Collective dataset earned a higher accuracy of 98.85% compared to the CASIA (v1.0) and CASIA (v2.0) datasets, which scored 98.49% and 98.25%, respectively. The model consistently achieves high True Positive Rate (TPR) values, indicating its ability to categorize spliced photos as genuine positives reliably. Due to continuously low false positive rate (FPR) values in various datasets, the rate of incorrectly classifying genuine photographs as spliced is minimal. When CASIA (v2.0) reaches a maximum of 99%, the AUC values, which indicate the overall ability to distinguish, remain consistently high. The findings demonstrate the SVM model's exceptional capability to detect manipulated images across many datasets.

**TABLE 2:** Performance evaluation of SVM in image‐splicing detection.

Dataset	Accuracy	True positive rate	False positive rate	AUROC
Collective	98.85%	99.62%	2.05%	0.97
CASIA (v1.0)	98.49%	99.23%	1.26%	0.97
DVMM	97.33%	97.69%	3.80%	0.96
CASIA (v2.0)	98.25%	97.23%	0.96%	0.99

### 4.2 Performance of the proposed method against different post‐processing attacks

Manipulating images involves using geometric transformations such as scale, rotation, and distortion, either individually or in combination, to conceal evidence of fraudulent activity. These modifications are associated with the transplanted region or regions in the CASIA (1.0) and CASIA (2.0) databases. The method's accuracy in response to these modifications is seen in Figure 5. Precise documentation of sharp edges (forged artifacts) is necessary when making geometric modifications at the boundary of a connected region (or regions). The DWT‐DRLBP descriptor can capture grafting artifacts, which is particularly helpful in cases with distinct geometric alterations.

**FIGURE 6:**
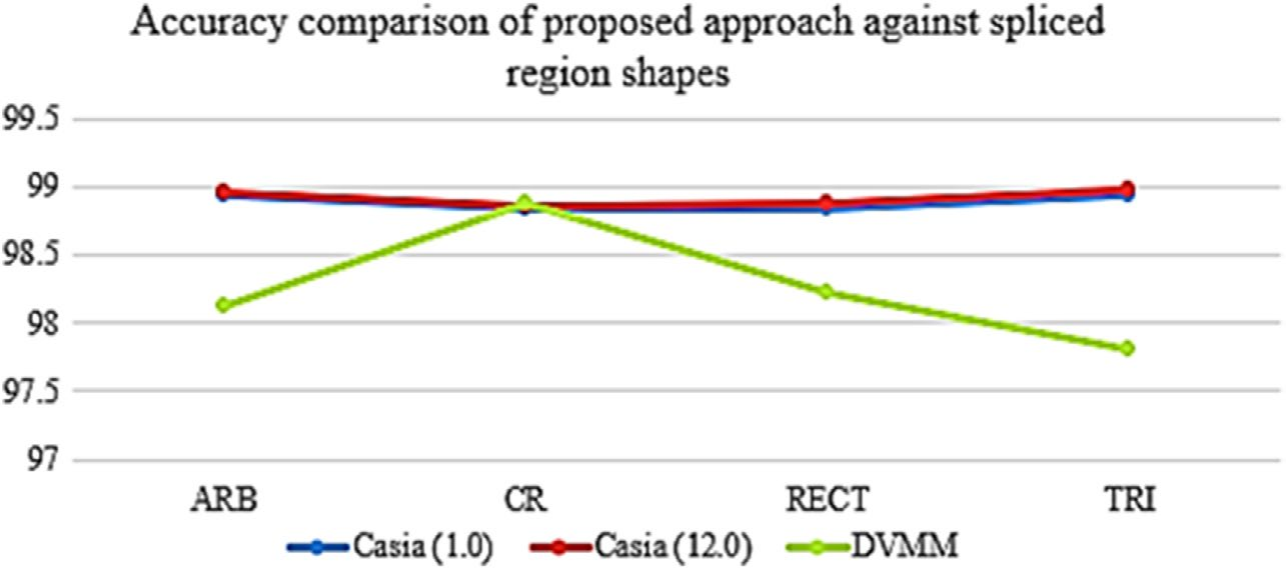
Accuracy comparison of spliced region shapes using the proposed detection method.

It is required to examine various intrinsic qualities, many of which are included in the original pictures, to detect similarities. Scientists analyze the interconnected regions of different dimensions using this method. The DTW‐DRLBP signifier has demonstrated its efficacy in detecting instances of imitation by effectively identifying localized abnormalities in the corresponding area or regions. Illegally amalgamating various forms to gain benefits is a widely seen behavior. This section examines the influence of interconnected geographical locations on performance. The CASIA datasets (versions 1.0 and 2.0) consist of circular, square, triangular, and rectangular variations of the connected part(s). Figure 6 illustrates the formations that have occurred consequently. Various types of grafted regions have a favorable response to this approach.

When the recommended technique was used on JPEG photographs from CASIA (1.0) and (2.0) datasets, it obtained an accuracy of 98.1%. DCT quantization can be used to compress JPEG files. The JPEG format can distort graffiti artwork and generate unattractive borders. Another contributing element to fraud is the failure to align squares with their neighboring squares. The provided approaches obtained exceptional accuracy in imitating by effectively examining the abnormalities in the combined picture utilizing the DWT‐DRLBP descriptor.

### 4.3 Comparative analysis with SOTA methods

This work assesses the validity and performance of the procedure by means of a comparison with top‐notch techniques from current investigations, therefore, determining their degree of success. Table 3 shows the suggested image‐splicing detection system next to a list of more sophisticated techniques together with their respective accuracy rates. Among the methods under assessment are SVM, DRLBP, and DWT. Evaluated on numerous datasets, including CASIA v1.0 and CASIA v2.0, the proposed method demonstrates astonishing accuracy rates of 98.48% and 98.24%, respectively. Especially, it highlights its efficiency since it offers an overall success rate of 98.85% over all datasets.

**TABLE 3:** Comparison with state‐of‐the‐art methods.

Approaches	Method	Dataset	Accuracy
Proposed	DWT + DRLBP + SVM	CASIA v (1.0)	98.48%
		DVMM	97.32%
		CASIA v (2.0)	98.24%
		All	98.85%
[36], 2023	Transfer Learning (VGG 16)	CASIA v (2.0)	95%
[37], 2024	ResNet	CASIA v (2.0)	92.50%
[30], 2020	CNN	CASIA v (1.0)	96.14%
		CASIA v (2.0)	93.86%
[7], 2023	DLBP + SVM	CASIA v (2.0)	97.8%
[38], 2023	CNN	CASIA v (2.0)	92.10%

The transfer learning approach employing VGG16 on CASIA v2.0 got 95% right, compared to the ResNet‐based method, which got 92.50% accurate. On CASIA v1.0 and CASIA v2.0, respectively, the CNN technique produced accuracy rates of 96.14% and 93.86%. Combining DLBP with SVM, another method attained an accuracy of 97.8% on CASIA v2.0. Applying a CNN‐based method to CASIA v2.0 yielded an accuracy of 92.10%. The proposed method outperforms existing techniques, improving accuracy across multiple datasets. Using DWT, DRLBP, and SVM together to find image splicing has also worked well, making progress in image forensics and security. The integration of many techniques, including DWT and DRLBP, with SVM for classification enhances the detection of picture splicing, as seen by the obtained findings. The suggested approach has consistently received excellent accuracy across many versions of the CASIA dataset, indicating its resilience and potential for application to additional datasets. The proposed approach outperforms transfer‐learning techniques utilizing VGG16 and ResNet‐based algorithms by delivering comparable levels of accuracy. The suggested technique exhibits superior accuracy compared to standard CNN‐based algorithms and DLBP paired with SVM. The findings emphasize the need for a comprehensive approach incorporating several attributes and classifiers to identify picture splicing effectively. These developments are essential to halt the spread of manipulated photographs and ensure the authenticity and integrity of all visual material [39, 40].

## 5 CONCLUSION AND FUTURE WORK

During the verification process, this research utilized the DWT‐DRLBP signifier to extract relevant information from the test picture. To exploit differences in grafting, the chroma modules of the sample picture are reduced in resolution to sub‐bands using a single‐level discrete wavelet transform (DWT). The sub‐bands provide intricate data that is acquired using DRLBP, a very efficient surface descriptor. By utilizing the DWT‐DRLBP descriptor, the proposed method may detect fake composite pictures in real‐world scenarios before post‐processing. The performance of the DWT‐DRLBP feature extractor is determined and assessed using a support vector machine classifier and a 10‐fold cross validation. The assessments and testing were conducted using three publicly accessible datasets. The strategy we suggested attained a 98.85% accuracy rate on the combined dataset, surpassing the accuracy of existing state‐of‐the‐art approaches. These results further support the notion that the DWT‐DRLBP descriptor effectively depicts the distortions caused by imitative behavior, and due to its ability to determine the relationship between two pictures, SVM with RBF yielded exact outcomes. Current efforts are concentrated on identifying the linked sites or areas to ensure consistency in the number of connections and increase trust in the results.

## FUNDING INFORMATION

Financial or technical support is provided by VTT Technical Research Centre of Finland and MLC Research Lab, Okara, Pakistan.

## CONFLICT OF INTEREST STATEMENT

The authors declare that they have no known competing financial interests or personal relationships that could have appeared to influence the work reported in this paper.

## Data Availability

The code and datasets will be available upon request to the corresponding author.
